# A Blueprint for Building a Renal Denervation Program

**DOI:** 10.1016/j.jscai.2025.104008

**Published:** 2025-10-21

**Authors:** Tayyab Shah, Catherine Vanchiere, Maria Bonanni, Debbie L. Cohen, Jay Giri, Taisei Kobayashi, Brian Fulton

**Affiliations:** a Division of Cardiovascular Medicine, Hospital of the University of Pennsylvania, Philadelphia, Pennsylvania; b Division of Renal, Electrolyte and Hypertension, Hospital of the University of Pennsylvania, Philadelphia, Pennsylvania; c Division of Cardiovascular Medicine, Chester County Hospital, West Chester, Pennsylvania

**Keywords:** blueprint, early career, fellows-in-training, renal denervation

## Abstract

Renal denervation (RDN) has emerged as a promising adjunctive therapy for hypertension, particularly in patients with poor blood pressure control despite pharmacologic and lifestyle interventions. With the recent approval of 2 RDN systems by the US Food and Drug Administration, there is growing opportunity for interested interventional cardiologists to develop expertise in this rapidly evolving, unsaturated field. This review serves as a comprehensive guide to building an RDN program, covering team development, training requirements, workflow design, and economic considerations. Interventional cardiologists, particularly fellows-in-training and those at early career stages, are uniquely positioned to advance this emerging technology by leveraging their multidisciplinary training, technological fluency, and openness to innovation to shape the future of interventional hypertension care. This blueprint offers a practical roadmap to capitalize on the clinical and career opportunities that RDN presents.

## Introduction

Hypertension (HTN) is the leading cause of cardiovascular disease in the United States, with an estimated prevalence among adults of almost 50%.^1^ Due to medication nonadherence, medication side effects, clinical inertia, patient specific factors (financial, cultural, psychological) and true resistant HTN (up to 20% of treated HTN population), less than one-fourth of these patients meet the guideline recommended blood pressure (BP) goal of <130/80.^2^ Over the past decade, renal denervation (RDN) has emerged as a promising interventional treatment for HTN in both patients off antihypertensives and those on antihypertensives without adequate response.^3^, ^4^, ^5^, ^6^

Renal denervation works by destroying the sympathetic nerves around the adventitia of the renal arteries, which are implicated in activation of the renin-angiotensin-aldosterone system, sodium retention, and increases in renal artery resistance. Although early studies on RDN yielded mixed results, advancements in device technology, refined patient selection criteria, and improved trial designs have now resulted in multiple sham-controlled randomized trials demonstrating the safety and efficacy of RDN devices.^2^^,^^7^, ^8^, ^9^, ^10^, ^11^ RDN decreases systolic BP by a mean of 5 to 10 mm Hg, which is comparable to the average BP reduction in placebo-controlled antihypertensive drug trials.^12^ In addition, up to one-fourth of RDN patients have decrements >10 mm Hg, and up to 10% have decrements >20 mm Hg.^2^^,^^11^^,^^13^, ^14^, ^15^, ^16^ Similarly, RDN increases the number of patients meeting BP goals without medications (absolute increase of 10%-20%).^13^^,^^14^ Further, although it is generally possible to achieve intensive BP control with medications, the landmark Systolic Blood Pressure Intervention Trial (SPRINT) showed that after the trial’s completion, most patients who had achieved BP control returned to baseline BP and lost the cardiovascular benefit of intensive BP control.^17^ RDN may offer a more durable solution in comparison to relying solely on patient adherence, given the lasting reductions in BP seen up to 3 years post-RDN,^3^^,^^15^^,^^18^ and it can double the time in therapeutic range,^15^ which is associated with 10% to 15% reductions in myocardial infarction, stroke, and cardiac death.^19^

As of November 2023, 2 RDN systems—the Symplicity Spyral system (Medtronic) and the Paradise system (Recor Medical)—have been approved by the US Food and Drug Administration (FDA) in patients whose BP is not well controlled despite lifestyle modifications and medications. Despite being a safe and straightforward procedure for appropriately-trained operators,^7^^,^^9^ the widespread adoption of RDN over the past year has been limited by issues regarding reimbursement and cost.^20^ The Centers for Medicare and Medicaid Services (CMS) released the proposed national coverage determination (NCD) decision for public comment in July 2025, with the final decision expected later this year. Thus, RDN offers interested interventional cardiologists, particularly fellows-in-training and early career cardiologists, a niche field that is not already saturated to jumpstart their careers. This comprehensive review outlines considerations for establishing a successful comprehensive RDN program, encompassing team assembly, patient workflow (before, during, and post-RDN), equipment acquisition and training, and financial planning.

## Establishing an RDN program within a multidisciplinary team

The foundation of an effective RDN program lies in assembling a team able to perform comprehensive preprocedural evaluations to ensure appropriate candidacy for the procedure. Ideally, this will take the form of a multidisciplinary team (MDT) dedicated to comprehensive HTN management (Central Illustration) as has been recommended by multiple cardiology societies.^7^^,^^21^^,^^22^ These teams have the goal of efficient and effective HTN management for the general HTN population and have the expertise to identify appropriate candidates for RDN and have access to the patient population that will serve as a robust source of referrals.^2^^,^^7^^,^^9^ Many institutions already have such a program and merely require the addition of an RDN proceduralist. Still, although the number of institutions with these teams is increasing, they remain in the minority.^23^ For those needing to create/refine a program, the team should be able to readily measure and follow-up serial BP measurements (office, home, or ambulatory monitoring) in a timely manner, be able to provide comprehensive lifestyle recommendations, escalate antihypertensive medications and monitor for side effects, perform appropriate testing to rule out secondary causes of HTN (and treat if a cause is identified), and perform and interpret renal vascular imaging (to rule out procedural contraindications such as renal artery aneurysm/stenosis/stenting or fibromuscular dysplasia).^7^ This will help ensure that the institution has the necessary personnel and infrastructure to identify appropriate patients for RDN.

**Central Illustration:**
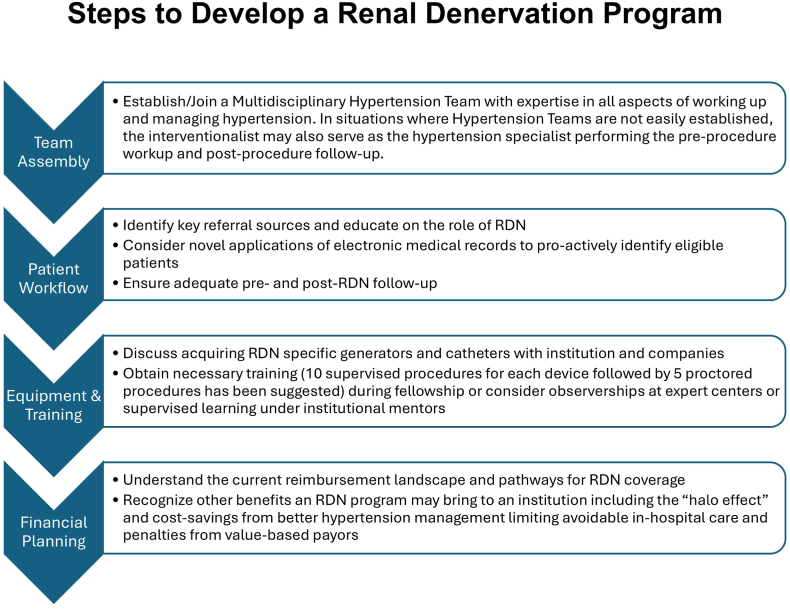
**Steps to develop a renal denervation program.** RDN, renal denervation program.

The key members of the MDT running the HTN program may vary by institution, but should include stakeholders from both invasive and noninvasive specialties. Potentially relevant specialties include primary care, general cardiology, nephrology, endocrinology, and the procedural subspecialty performing RDN at the given institution (interventional cardiology, interventional radiology, vascular surgery, etc).^7^ At the center of the comprehensive HTN team should be the “hypertension expert” who not only has the expertise but can also coordinate care with proceduralists. At our institution, the MDT includes nephrologists and cardiologists as well as advanced practice providers (APP). Some institutions also incorporate pharmacists. The APPs have been invaluable to our program and are key members of the team, allowing for expeditious follow-up, remote monitoring of BP, timely titration of BP medications, completion of pre-RDN testing even before referral to the proceduralist, and completion of prior authorizations for medications and procedures. In the early stages of program development, such a comprehensive team is not absolutely necessary, but would require that the interventionalist become the HTN expert and lead these efforts in their own clinic using available local resources. In these cases, it will also be vital that the interventionalist ensures they have expertise in the comprehensive workup and management of HTN or identify key stakeholders who do.

As an example, at our institution, the first visit is with a HTN expert, who may be a noninvasive cardiologist, nephrologist, or APP specifically trained in HTN management. At that visit, care will be taken to ensure an accurate BP measurement, ideally using an automated office BP cuff after resting alone for 5 minutes. A 24-hour ambulatory BP monitor is typically completed to confirm out-of-office BP control or lack thereof. A comprehensive workup is then initiated in appropriate patients with imaging and lab testing to evaluate for secondary causes of HTN, including primary hyperaldosteronism, renovascular disease, drug- or supplement-induced HTN, obstructive sleep apnea, and certain neoplasms, as well as rarer causes based on individual patient factors.^24^ At our center, APPs complete this process independently. Although exact practice may vary by location, it is important to create specific institutional protocols and pathways to improve efficiency and timeliness.

Incorporation of APPs^25^, ^26^, ^27^ and pharmacists^28^^,^^29^ into the MDT for the management of chronic diseases, including HTN has been shown to improve access, quality, and cost. Incorporating dedicated APPs in the HTN program will be particularly important when starting a RDN program, given they will be able to assist with care, including the coordination of studies or tests mandated by payors based upon their coverage determinations.

Finally, although not a necessity, the Society for Cardiovascular Angiography & Interventions (SCAI) recommends that HTN programs considering RDN obtain American Heart Association Hypertension Center Certification.^7^ This offers interventionalists establishing new HTN programs a checklist of services and often relies on choosing the right partners in the MDT to provide a full suite of HTN therapies.^30^ Still, inability to obtain this certification, particularly in the early stages of program development, should not discourage interested institutions from starting an RDN treatment program.

## Obtaining necessary equipment and training

Renal denervation procedures require device-specific generators and catheters. However, most other equipment should already be available in most cardiac catheterization laboratories. At present, all RDN technologies require femoral access. As such, shorter 55 cm guiding catheters (internal mammary artery or renal double curve most frequently, and the multipurpose or Amplatz shapes less frequently) are utilized. As the technology naturally evolves toward radial access, we expect that there will be more reliance on the multipurpose catheter. Guide wires should be 0.014" nonhydrophilic workhorse wires.^31^ In the rare event of renal artery complications such as stenosis, dissection, or rupture, operators must also have access to covered and noncovered stents and/or coils for the renal artery.^7^^,^^9^

SCAI has recently released guidance on training and competency for RDN procedures.^7^^,^^32^ Historically, when renal artery interventions were more common, it was recommended that operators had done at least 30 diagnostic procedures and 15 interventions with at least half as the primary operator. However, given the much lower rates of renovascular interventions in contemporary practice and the relative safety of RDN, SCAI has relaxed these suggestions. First, dedicated advanced endovascular training is not required to perform RDN, given that the procedure in contemporary trials was performed safely and effectively by many operators without previous renovascular experience. For operators interested in performing RDN without advanced endovascular training, SCAI recommends a number of training pathways to obtain the necessary skillsets, including didactic/simulation modules, procedural observerships at RDN centers, or supervision from an experienced renovascular operator at their current institution. Further, SCAI recommends 10 supervised procedures with each approved device for inexperienced operators before moving on to a proctored phase where an expert operator or company representative proctors at least 5 cases to ensure operator competency. Among endovascular-trained operators with endovascular privileges, SCAI recommends moving directly to the proctored cases.

## Patient referral

Once the necessary infrastructure, personnel, and equipment are in place, institutions are ready to offer RDN treatments as an option for uncontrolled HTN. At our center, we utilize both in-reach and outreach initiatives to inform and educate referring providers on the role of RDN in the treatment of HTN. Although many patients are referred through conventional pathways, it is conceivable to leverage modern-day electronic medical records to extract data of those who demonstrate uncontrolled HTN.^33^ For example, automation, artificial intelligence, and machine learning applications identifying and funneling patients to the HTN center are currently being developed at our institution.

Prior to referral, the proceduralist should discuss the RDN procedure with patients, including risks/benefits, follow-up requirements, and anticipated results. Although every discussion will be slightly different, interventionalists should note that, based on surveys, some patients and providers may have unrealistic expectations on the efficacy of RDN,^2^ and it is vital to set appropriate expectations to increase the likelihood of patient and referring provider satisfaction. As an example, in our practice, we explain to patients and referring providers that BP changes associated with RDN may not manifest for months postprocedure, will be comparable to 1 additional antihypertensive medication, and up to one-third of patients may not respond to RDN.^2^^,^^7^, ^8^, ^9^, ^10^, ^11^ Additionally, as RDN becomes more commonplace, it is important not to become overexuberant with its use in populations that lack evidence of benefit, as often occurs with new technologies. Centers should develop more experience and expertise in managing RDN patients before considering its use in unstudied populations.

## Procedural management

The 2 available RDN systems—the Symplicity Spyral system (Medtronic) and the Paradise system (Recor Medical) share similar safety and efficacy profiles, but they employ different mechanisms of action—radiofrequency ablation versus ultrasound energy, respectively—and differ in procedural nuances such as catheter design, ablation duration, and recommended technique. A detailed comparison of these systems, including device specifications and procedural steps, is beyond the scope of this review and is summarized in existing literature.^31^ Briefly, both procedures are performed via femoral arterial access using standard 6 to 7F sheaths. Renal angiography is performed at the outset of each case, even if high-quality cross-sectional imaging was done preprocedurally. This step confirms the number and location of renal arteries, identifies accessory arteries (which may be amenable to ablation), and ensures that the vessels are free of anatomic contraindications such as severe atherosclerosis, renal artery stenosis, dissection, or fibromuscular dysplasia. Once selective angiography is performed, a 0.014" nonhydrophilic guide wire is delivered through the 55 cm guide into the renal arteries. The RDN catheter is advanced, and areas of the artery between 3 and 8 mm are denervated; accessory renal arteries within the target diameter range should also be treated. Particular care must be taken to avoid ablating within or adjacent to renal artery stents or heavily calcified plaque, where thermal energy delivery may be ineffective or risky. In both technologies, a distal to proximal treatment strategy should be employed. The Symplicity Spyral system applies segmental, circumferential radiofrequency ablations in both the main renal artery and distal branches. In contrast, the Paradise system delivers circumferential ultrasound energy via a balloon catheter that is inflated within the main renal artery and cooled with circulating water.^31^ Before ablation begins, the use of moderate to deep sedation or monitored anesthesia care is vital, given the notable pain associated with renal nerve denervation.

Intraprocedural complications are exceedingly rare, with most complications related to femoral access, and significantly <1% of patients experiencing renal artery complications (dissection, rupture, spasm, stenosis).^34^ Still, operators must be prepared to identify and manage any complications. Femoral artery complications should be handled using standard local practices employed for other femoral artery based procedures including percutaneous coronary intervention. Among renal artery complications, the most common finding is renal artery spasm, which typically presents as transient vessel narrowing without flow limitation and responds readily to intraarterial vasodilators (eg, nitroglycerin or nicardipine). A related but distinct angiographic finding is “notching,” which refers to subtle contour irregularities or small invaginations in the arterial wall, often seen post-ablation. These are typically benign and self-limited. In a very small subset of patients, renal artery dissection or perforation from the guide catheter, wire, or device can occur. True renal artery dissection may present with persistent linear defects, abrupt luminal tapering, and delayed flow, but has not been reported in the randomized control trials of these technologies. If confirmed, management depends on severity: minor dissections may resolve with observation and medical management; significant dissections may require renal artery stenting (typically with a bare-metal or covered stent). Guidance on renal artery stenting has been published separately.^35^ In the rare event of perforation, endovascular bailout with a covered stent or coil embolization may be necessary depending on the site of the perforation, particularly if there is extravasation or hemodynamic instability. Attention to wire positioning during the procedure should avoid distal wire perforations. Should it occur, this can lead to renal capsular hematoma which may present as postprocedural flank pain and kidney failure.^36^^,^^37^ However, there are no reported cases in the RDN literature.

Other procedural considerations include the use of intravenous hydration and judicious contrast use, especially in patients with chronic kidney disease. Most patients can be discharged on the same day after a period of hemostasis and observation, mirroring current same day percutaneous coronary intervention protocols.^7^

## Postprocedural management

**Figure 1:**
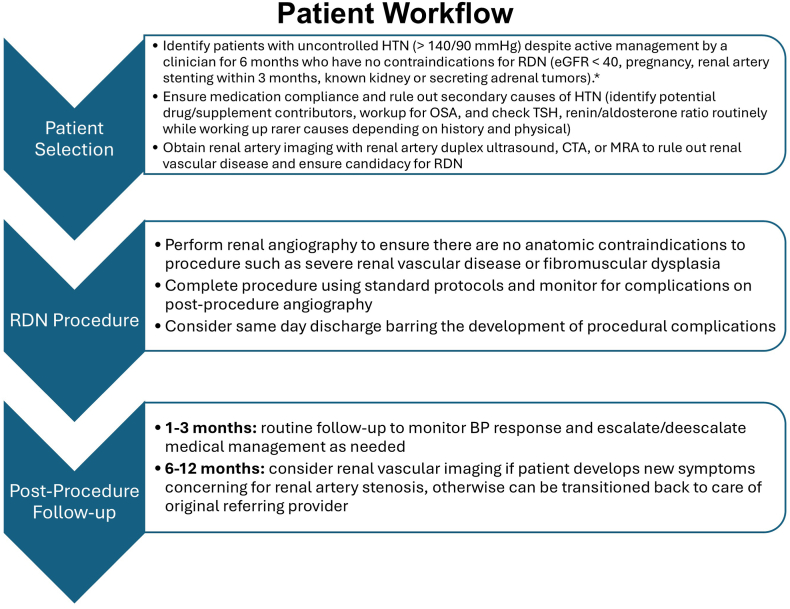
**Patient workflow from preprocedural to postprocedural management.** ∗Indications and contraindications listed per the recently proposed National Coverage Determination by the Centers for Medicare and Medicaid Services and may be subject to change in the final document. BP, blood pressure; CTA, computed tomography angiography; eGFR, estimated glomerular filtration rate; HTN, hypertension; MRA, mineralocorticoid receptor antagonist; OSA, obstructive sleep apnea; RDN, renal denervation; TSH, thyroid stimulating hormone.

Although current research protocols for ongoing postmarket approval studies have had follow-up visits at 1, 3, 6, and 12 months postprocedure, these are not mandated by the FDA. As noted above, these clinical trials have demonstrated the strong safety profile of RDN, with renal artery complications occurring no more frequently in the RDN population relative to controls,^2^^,^^11^ and SCAI does not advocate for any routine postprocedural imaging.^7^ Thus, postprocedure follow-up by the interventionalist and timing of transitioning care back to the referring provider or HTN clinic (generally within 6-12 months) may vary depending on local practices and patient specific factors, including immediate procedural complications or symptoms suggestive of renal artery stenosis. It should be noted that in the rare cases that renal artery stenosis does occur, it occurs within 6 to 12 months post-RDN procedure.^34^ A possible timeline from preprocedural to postprocedural management is presented in Figure 1. The timeline is largely consistent with the current proposed NCD by CMS; however, the NCD is open for public comment and may change before the final coverage decision is enacted. RDN programs should note that the current iteration of the NCD mandates intensive preprocedural follow-up and a patient coordinator, but specifics of frequency and necessity for a patient coordinator may be subject to change in the final iteration of the NCD.

Postprocedural management should focus on ensuring the absence of procedural complications and ensuring adequate medical management of HTN, which could include escalation of HTN medications in nonresponders and deescalation in responders. Importantly redo RDN procedures have not been studied.

## Financial considerations

Interventionalists building a comprehensive HTN program, including RDN, must be aware of the shifting landscape of the economics of RDN to appropriately leverage resources and gain institutional buy-in. At least 1 study using the contemporary randomized data has demonstrated that RDN is highly cost effective based on generally accepted United States thresholds (an incremental cost-effectiveness ratio of <$50,000 per quality-adjusted life year is generally considered highly cost effective, with the ratio being $32,732 for RDN).^38^ However, this cost-effectiveness has not yet translated into adequate reimbursement by payors. Thus, while we await the final results of the ongoing CMS review of NCD for RDN (with the final decision expected later this year and the earliest effective date of 2026), interested interventionalists must understand the currently available reimbursement and other potential benefits an RDN program brings to an institution in order to advocate effectively.

Both FDA-approved devices require not only a capital purchase of a generator but also the consumable products of the catheter(s). At present, RDN is mapped to category III temporary current procedural technology (CPT) codes (0338T and 0339T), primarily designated for emerging technologies/research contexts. CPT codes assign relative value units to each procedure, which are linked, but not directly, to payment. The CPT code maps to a level 2 endovascular procedure ambulatory payment classification (APC) code that reimburses an average unadjusted rate of $5452. APC codes are assigned by CMS and range from level 1 to 4, with higher numbers corresponding to more complex procedures with higher reimbursement. It is expected that RDN will transition to a category I CPT code, which is reserved for broadly used interventions with proven efficacy, and could standardize billing and subsequent reimbursement for RDN. It remains to be seen if this will also correspond with an upgrade in the APC code to level 3 or 4, which would likely be required to make the procedure profitable.

In the interim, effective October 2024, CMS will pay new technology add-on payments through the Medicare Hospital Inpatient Prospective Payment System^39^ for both approved RDN devices. Additionally, CMS has also approved transitional pass-through payments for both devices through the 2025 Medical Hospital Outpatient Prospective Payment System.^40^ These were effective as of January 1, 2025, and will be in place for 3 years; although it will assist hospitals in covering the cost of RDN cases, it will likely not be enough to cover the entire cost of RDN. In theory, Medicare Administrative Contractors could make local coverage determinations in the absence of a national determination; however, to our knowledge, no contractor has provided additional reimbursement for RDN.

It is important to know that several other technologies also went through these growing pains, including transcatheter aortic valve replacement (TAVR), where these procedures were loss leaders for institutions adopting this technology early. Echoing the early experiences for TAVR programs—where the availability of TAVR enticed more patients to seek care at TAVR-capable institutions, thereby increasing volumes of related procedures, including echocardiograms, imaging studies, cardiac catheterizations, and even surgical aortic valve replacement^41^—this “halo effect” of an HTN/RDN program must be considered when considering resource allocation. This is particularly relevant for RDN, given the high prevalence of HTN and the widespread demand for RDN among hypertensive patients (with about one-third of patients with HTN saying they would undergo RDN).^2^^,^^42^ Thus, an RDN program has the potential to bring hundreds, if not thousands, of new patients to an institution, all of whom will require multiple imaging tests prior to any procedure and many of whom have comorbidities requiring other treatments and/or procedures unrelated to RDN. This new technology gives interested interventionalists the opportunity to attract new patients and generate sustainable and productive practice models to allow for not only programmatic but also professional growth in an unsaturated field of interventional therapies for HTN.

Additionally, as noted above, RDN can substantially reduce BP in some patients and increase the time patients are in the time in therapeutic range.^2^^,^^11^^,^^13^, ^14^, ^15^, ^16^ It is vital to consider how this can also result in cost-savings to institutions when negotiating for resources. Although there are limited data on the effect of RDN on hard cardiovascular outcomes,^19^ it is reasonable to assume its effects on BP will have similar benefits to medications in reducing cardiovascular admissions/readmissions related to HTN and associated conditions, which can be costly to hospitals. Indeed, about 1% of all emergency room visits and 13% of all cardiac-related emergency room visits in the United States have a primary diagnosis of HTN, most of which are not related to complex complications of HTN but rather basic HTN management that do not lead to admissions.^43^, ^44^, ^45^ Improved outpatient management of HTN may help hospitals avoid these costly and unnecessary emergency room visits. Moreover, multiple programs through CMS, including value-based purchasing, accountable care organizations, and pay-for-performance programs, and similar programs from private insurers link reimbursement bonuses and penalties with quality (with performance metrics generally including outpatient HTN control).^46^, ^47^, ^48^ Operators should gain an understanding of how their institution is reimbursed by CMS and private insurers, as this may be another avenue through which institutions can benefit from RDN programs by improving HTN control in their patient population, which is generally dismal across the United States.

## Conclusion

Following the FDA approval for commercial use of RDN technologies and a final decision regarding NCD by CMS in the coming year, we expect to see an exponential growth in the number of patients undergoing RDN. Building a successful RDN program is feasible for providers of all career stages when the appropriate steps are taken to build an MDT, establish/integrate into institutional HTN programs, obtain necessary equipment and training, determine protocols for preprocedural and postprocedural care, and gain institutional buy-in by understanding the current economics of RDN. Interventionalists, with their modern training in multidisciplinary care, openness to novel approaches to treatment of cardiovascular disease, and commitment to further conduct research into device technology and patient outcomes, are well-positioned to succeed as leaders in the development of this field.
